# Maryland State Dental Association

**Published:** 1892-05

**Authors:** 


### Editorial, Etc.

MARYLAND STATE DENTAL ASSOCIATION.—The Maryland State Dental Association, organized in 1883, held its ninth annual session in the St. James Hotel, Baltimore, May 9 and 10, 1892. Annual meetings have been held without interruption. In 1887 it had but seven members in good standing, but after that time interest in the organization increased, and upon its absorbing the State Odontological Society, a rival body, its membership numbered in 1889 about ninety dentists. The meeting in 1889 was conspicuous for the able papers read and for the harmony that prevailed in all the proceedings. The same may be said of the session just closed. Two business matters of especial interest to the profession at large were adopted; one, appointing a committee of eight members to convey to the Senators and Representatives of Maryland in Congress the protest of the dentists "against the action of the Census Bureau in classifying dentists as manufacturers", and the other the appointment of a committee of three to confer with a similar committee from the State Board of Dental Examiners, and draft a dental bill to be presented with their endorsement to the Legislature in 1894.

The Committee on Operative Dentistry noted some adverse criticism against the report of its predecessor in 1891, which "advocated so strongly the practice of extracting the sixth-year molar," and its report said that "the acceptance of the report, as a whole, caused a notice to appear subsequently in the *Dental Cosmos* to the effect that the Association endorsed, among other recommendations. the extraction of the sixth-year molars.'' To avoid such a construction as to the papers read at the ninth session there is printed with the proceedings the resolution adopted December 6, 1889; to wit, "This Association is not responsible for the views of the authors of the papers published."

